# New horizons in criminal legal data: creating a comprehensive archive

**DOI:** 10.1186/s40352-024-00286-5

**Published:** 2024-07-17

**Authors:** Katherine LeMasters, Erin McCauley, Lauren Brinkley-Rubinstein

**Affiliations:** 1 Division of General Internal Medicine, University of Colorado Anschutz School of Medicine, Aurora, CO USA; 2 Department of Social and Behavioral Sciences, University of California San Francisco, San Francisco, CA USA; 3 Philip R. Lee Institute for Health Policy Studies, University of California San Francisco, San Francisco, CA USA; 4 Department of Population Health Sciences, School of Medicine, Duke University, Durham, NC USA; 5 Division of General Internal Medicine, University of Colorado School of Medicine, 8th Floor, Academic Office 1, Mailstop B180, 12631 E 17th Ave, 80045 Aurora, CO USA

**Keywords:** Criminal legal data, Health equity, Data archive

## Abstract

While criminal legal involvement is a structural determinant of health, both administrative and national longitudinal cohort data are collected and made available in a way that prevents a full understanding of this relationship. Administrative data are both collected and overseen by the same entity and are incomplete, delayed, and/or uninterpretable. Cohort data often only ask these questions to the most vulnerable, and do not include all types of criminal legal involvement, when this involvement occurs in someone’s life, or family and community involvement. To achieve a more optimized data landscape and to facilitate population-level research on criminal legal involvement and health, (1) individual administrative level data must be made available and able to be linked across carceral systems, (2) a national data archive must be made to maintain and make criminal legal data available to researchers, and (3) a nationally representative, longitudinal study focused on those with criminal legal involvement is necessary. By beginning to critically think about how future data could be collated and collected, we can begin to provide more robust evidence around how the criminal legal system impacts the health of our society and, in turn, create policy reform.

There are well-established connections between criminal legal involvement and health (Wildeman & Wang, 2017). Yet, the arbiters of administrative criminal legal data (e.g., Bureau of Justice Statistics (BJS) and existing national longitudinal cohort data (e.g., National Longitudinal Survey on Youth) collect and share data in such a way that prevents population-level criminal legal involvement from being fully documented in a timely and accessible way. For example, administrative data on arrests are collected and shared separately from data on jails, which are collected and shared separately from data on probation, making a full understanding of the system’s scope impossible. Additionally, cohort data often ask questions in such a way that misses pre-trial detention (e.g., only asking about jail incarceration after sentencing), even though 83% of those incarcerated in United States jails are in pre-trial detention and are not convicted of a crime (Sawyer, W & Wagner, P, 2023). These incomplete data prevent the relationship between the criminal legal system and health outcomes from being fully understood, which has been long-documented (Byrne et al., 2008). Criminal legal data being standardized and made available in real-time is a first, imperative step towards fully understanding the relationship between the criminal legal system and health. The current paper is a discussion of current limitations in criminal legal data and opportunities for collating and collecting more robust data.

## Administrative criminal legal data: incomplete, delayed, and uninterpretable

Administrative data are data which are collected by organizations and government entities for program administration, regulation, or enforcement. Although not collected for research purposes, these data hold great promise for research because they are at the individual level, reflect whole populations, and are already collected. Criminal legal system administrative data are owned by and housed in the BJS within the Department of Justice (U.S. Office of Management and Budget, 2017). This presents three issues. The first is related to purview: the only data published are the data that the BJS collects. This does not include, for example, data on policing, including police-related death, or arrests. The second issue is related to timeliness: the BJS publishes death data at a multi-year delay, preventing a real-time response. The BJS also recently stopped aggregating data on deaths in custody altogether, thus preventing any public health response (Carson, 2021). The third issue is related to interpretability and transparency: The BJS is housed under the Department of Justice, which oversees the criminal legal system. This means that data are likely incomplete (e.g., undercounting deaths in custody) and there is little incentive to improve data. For example, while suicide deaths - deaths that carceral entities play an outsized role in preventing - are reported from BJS, investigative reporting has found them to be a large undercount in Georgia, a pattern that likely holds true for other state systems (LeMasters et al., 2023; Robbins, D & Peebles, J, 2022).

There has been growing attention to the lack of timely, comprehensive, and standardized data about the criminal legal system. For example, the Justice Center’s Justice Counts Initiative provides tools, resources, and support for agencies to share data within the criminal legal system. Outside of the criminal legal system, The Third City Project investigates each of the United States 53 Departments of Corrections’ death reporting (Third City Project, 2023). The Third City Project documented that only 29 systems report any individual-level death data, and almost none report complete data (e.g., decedent’s race, description of death), or report deaths in a timely manner (e.g., within three months of them occurring) (Fliss et al., 2024). This reporting is incompatible with a public health approach to being able to understand and respond to deaths in custody, and it is in violation of the Federal Deaths in Custody Reporting Act (Behne, M et al., 2022). Furthermore, Congress introduced the Federal Prison Oversight Act in 2022 to conduct inspections of prisons and to establish an ombudsman in the Justice Department given the current lack of accountability (Federal Prison Oversight Act, 2022). This incomplete, delayed, and often uninterpretable data make it imperative that criminal legal data are archived by an independent entity outside of the BJS and fact-checked from a variety of sources for public health researchers and practitioners to understand life course trajectories of criminal legal involvement and to estimate causal effects of the criminal legal system on health at a population level.

A final limitation of the current administrative data available through BJS is that although administrative data are collected at the individual level, the BJS largely only release these data in aggregated form. This limits the utility of these data. The aggregated nature of the data makes it impossible to track individuals’ pathways through the system or to conduct research comparing how different types of involvement (e.g., arrest, probation) in different jurisdictions impact health. It is also important to be able to stratify current data by certain socio demographic groups. Currently, BJS aggregates data by gender and race only. This makes it impossible to stratify analyses by important variables such as disability status, and to stratify by multiple socio demographic characteristics at one time (e.g., how many Black women died in custody in a year). Furthermore, it makes it impossible to stratify by locale, which is critical given how local resources and policies affect both criminal legal involvement and its relationship to health.

## Nationally representative cohort criminal legal data

Longitudinal criminal legal data collected as part of national cohort study allow researchers to both follow someone’s trajectory in the system and to understand the complex ways that the legal system shapes the life course, but they too, often have limitations. For example, when national longitudinal surveys are created by public health experts that lack criminal legal expertise, surveys include criminal legal question categories that are not aligned with how the criminal legal system works. We now outline six limitations common to national longitudinal cohort data:

1. These questions often focus on prison incarceration, even though this is less common than other forms of criminal legal involvement (Sawyer, W & Wagner, P, 2023).There is also evidence that prison time is harmful for health, so the exposure of prison is often what public health researchers focus on (Wildeman & Wang, 2017). In fact, there are recommendations for better measuring criminal legal involvement from the National Academies of Medicine, but the focus remains on incarceration, the type of facilities someone is housed in, and crime (National Academies of Medicine, n.d.). This presents two main issues: (1) ignoring criminal legal involvement other than incarceration precludes a full understanding of total involvement and inequities in involvement (LeMasters et al., 2022); (2) a focus on crime rather than criminal legal involvement ignores the racialization of what is considered and reported as crime (McNamarah, 2019).
2. If questions do go beyond incarceration, they often provide mutually exclusive categories that are poorly aligned with how the system operates. For example, the National Longitudinal Survey on Youth only allows probation questions to be asked if individuals received no jail or prison sentence while The National Longitudinal Study of Adolescent to Adult Health only allows individuals to select that they were sentenced probation or jail or prison incarceration for given arrest (*Add Health Codebook Explorer*, n.d.; National Longitudinal Surveys, n.d.).
3. These questions are also not always standardized across survey waves, preventing a full understanding of individuals’ pathways through this system.
4. Many surveys also do not document the age at which the criminal legal encounter occurred. As these health-harming criminal legal encounters most frequently occur at young ages, it is critical to know when and how often they are occurring. Further, collecting age of first encounter would allow researchers to harness demographic methods to develop population estimates for a variety of sociodemographic groups (e.g., generate estimates of the lifetime risk of criminal legal involvement by gender, race, and disability status) (LeMasters et al., 2022). Accurate representations of how and when individuals become entangled in the criminal legal system would allow researchers to understand unique risk factors predating criminal legal involvement and trajectories of deeper system involvement for different groups.
5. Most surveys fail to capture involvement beyond the individual level. Surveys rarely ask sufficient questions about parental or family member incarceration even though 45% of individuals will experience the incarceration of a family member in the United States (Enns et al., 2019). And, surveys rarely capture neighborhood or community-level involvement, even though high levels of carceral involvement of those around you is known to be harmful for health (Hatzenbuehler et al., 2015; LeMasters et al., 2023b).
6. Lastly, these questions are most thoroughly asked in cohorts already focused on vulnerable populations, such as the Future of Families and Child Wellbeing Study (formerly known as Fragile Families), rather than all populations (Princeton University, n.d.), and tend to have poor retention.

These shortcomings shape the research questions being asked and explored and prevent a deeper understanding of individuals’ pathway through this system. These shortcomings also likely provide an underestimation of criminal legal involvement itself and an underestimation of inequities in involvement both within communities and over the life course of an individual. Prior work has outlined how research studies can better ask criminal legal questions to reduce error and missingness, including providing participants with a definition of criminal legal involvement (Yan & Cantor, 2019). We must also ensure that these questions are asked in the way that the system actually works (e.g., including questions beyond incarceration; allowing individuals to select experiencing jail and probation), are standardized across survey waves, capture ages of involvement, and capture family and neighborhood involvement. Critically, these questions must be asked to all populations, not only to cohorts focused on the most vulnerable.

## A three-part solution

While difficult to accomplish, it is important to think critically and strategically about what the future of criminal legal data could be. To this end, we propose three steps to achieve more optimized data to facilitate population-level research on criminal legal involvement and advance inquiry on the public health costs of mass incarceration.

First, individual level data are needed that could be linked across carceral systems. These data could be managed by the BJS itself, pre-established archives such as the Inter-university Consortium for Political and Social Research (ICPSR) or the Criminal Justice Administrative Records System (CJARS), or a new criminal legal data archive. These administrative data are already collected by various institutions for the provision of services, including probation departments (e.g., technical violations), county jails (e.g., substance use treatment receipt), state prisons (e.g., solitary confinement), and federal prisons, and could be made available for researchers. The exact measures available in these data would depend on what their respective systems already collect, as administrative data are already collected for service provision and would simply be collated and made usable for researchers. These data could be given a unique identifier and deidentified to protect privacy (e.g., recoding birthdates to the first of every month), making it possible to analyze data independently or to link individuals across pathways of criminal legal involvement. Ideally, these data could be collated across more systems through partnerships with data administrators including immigration detention and Tribal data administrators, as Native American tribes own their jail data, to ensure data are complete and accurate (Hudson et al., 2023).

Such comprehensive data with individual-level linkage capacity would require institutional buy-in (e.g., Bureau of Prisons), resources, and coordination at many levels. Yet, these administrative data are already collected, and institutions have a mandate to be accountable to the populations that they serve. Further, these data could revolutionize our understanding of how the criminal legal system works, how it shapes the life course of those entangled in it, and the broader costs of this system for our society. In many ways, this system, which is rooted in racism and oppression, has benefited from siloing its data and obscuring its health effects, thus conditioning public health to think of these systems and data as outside of our purview (Bailey et al., 2017; McCauley et al., 2023). In the context of this data desert, researchers have sought to challenge these systems and study their implications through building evidence through initiatives that collate these data such as The Third City Project (Third City Project, 2023). As we consider new horizons in data for our field, we must place pressure on carceral institutions to make these data available to research and look to other examples to how this type of data landscape change has been successful in the past.

Second, to improve transparency around criminal legal system data (e.g., accurately document in-custody deaths, which do not occur in the aforementioned example in Georgia) and foster the study of the health consequences of criminal legal system involvement, we call for the development of a national data archive which maintains and makes criminal legal data available to researchers. Ideally, this single outside entity would include the administrative data discussed in Point 1, as well as any other data related to the criminal legal system which researchers collect. Given the dire reporting on and current political momentum around deaths in custody (e.g., The 2022 Federal Prison Oversight Act), we recommend prioritizing collating these deaths (at an individual level and with sociodemographic information) as an important first step for a potential archive.

Such archives increase evidence by increasing the amount of research and publications, foster interdisciplinary collaboration, and preserve the history of systems. No official national repository other than the BJS exists relevant to criminal legal data (though some independent data collection occurs for specific types of criminal legal data - such as mortality data from The Third City Project and population data from ICPSR and CJARS). However, there are examples of how to expand what we know. Child welfare system data are archived with the National Data Archive on Child Abuse and Neglect (NDACAN) (*National Data Archive on Child Abuse and Neglect*, n.d.). This outside entity, funded by a contract with the Children’s Bureau, archives administrative data collected across multiple systems and databases (e.g., the National Youth in Transition Database) through establishing cooperative agreements, tying agencies’ funding to meeting reporting requirements, and using cohort-based studies collected by researchers. These administrative data are de-identified to protect children’s privacy and are cleaned to facilitate use by the research community. NDACAN also uses a unique child identifier, which allows researchers to trace individual children through Child Protective Services, the foster care system, and, for those who age out without finding a permanent placement, the transition to adulthood. NDACAN preserves the history of the system, undertaking historical data acquisition to uncover and digitize past administrative data. Lastly, NDACAN provides technical support to support users in conducting analyses with administrative data, promoting the use of these data within the child welfare research community, and promoting the study of child welfare more broadly.

While difficult, it is feasible to create an archive for data related to the criminal legal system. Ideally, the archive could compile data across policing, jail, prison, probation, parole, and immigration detention systems, and would include a unique identifier to track individuals through these systems. Additionally, these administrative data have already been collected - meaning that researchers will not further burden this population by asking about their criminal legal history and would avoid recall bias and underreporting. Further, researchers studying the criminal legal system across disciplines, including public health, sociology, economics, psychology, and history could have a central location to both access and deposit their data. This would allow us to increase the utility of data related to the criminal legal system by increasing the number of studies in which they are used – a recent study found that archiving data resulted in two-fold the number of publications – and reduce barriers to studying the societal costs of mass incarceration (Pienta, A et al., n.d.).

Third, a longitudinal, nationally representative study focused on criminal legal involvement is needed to fully capture carceral experiences across the life course and their effects on health. National longitudinal surveys exploring other topics, such as the National Longitudinal Study of Adolescent to Adult Health, the National Longitudinal Survey of Youth, and the Future of Families and Child Wellbeing Study, have advanced research in their respective fields (e.g., the long-term consequences of adolescent contexts and behavior, the lives of unmarried parent families, labor market trends) and had immeasurable positive benefits for the research community with a combined 15,500 publications (Harris & Halpern, 2022; James et al., 2021). A separate national longitudinal birth-cohort survey aimed at studying the myriad of ways that the criminal legal system shapes the health and wellbeing of individuals, families, and communities would allow researchers to address questions of great interest to researchers and policy makers, including (1) what are individuals’ pathways through the criminal legal system across the life course, (2) how do carceral policies affect individual and community health, (3) how might certain types of exposure (e.g., jails versus prisons) and order of exposure differentially affect health outcomes, and (4) how does volume of criminal legal exposure impact health and structural determinants of health (e.g., housing stability)? Beyond the immediate benefits of this study to understand the complex ways that system involvement alters the life course of individuals and their families, having a national longitudinal survey designed to study this topic would create a standard in how we ask criminal legal questions for comparison. Finally, the availability of such robust and comprehensive longitudinal data would encourage researchers across disciplines to explore the consequences of the criminal legal system across a variety of spheres in addition to health.

## Conclusion

Current administrative and national longitudinal cohort data on the criminal legal system are insufficient, prohibiting a full understanding of how the carceral system affects health at the individual and community level. However, we have the tools to improve this understanding. We must focus on (1) improving individual-level administrative data so that research can link individuals through multiple criminal legal-related systems, (2) establishing a data archive to improve data accuracy and accessibility, and (3) creating a longitudinal, nationally representative cohort study focused on criminal legal involvement and health. By outlining these priorities for criminal legal data, we hope to make clear the potential for future data to generate change. With these changes in data collation and data collection, we can provide comprehensive evidence around how the criminal legal system impacts individuals, families, and communities and how it is affecting the health of our society. In turn, we can create evidence for policy reform.

## Data Availability

Not applicable.
